# The Lectin LecB Induces Patches with Basolateral Characteristics at the Apical Membrane to Promote Pseudomonas aeruginosa Host Cell Invasion

**DOI:** 10.1128/mbio.00819-22

**Published:** 2022-05-02

**Authors:** Roland Thuenauer, Katja Kühn, Yubing Guo, Fruzsina Kotsis, Maokai Xu, Anne Trefzer, Silke Altmann, Sarah Wehrum, Najmeh Heshmatpour, Brian Faust, Alessia Landi, Britta Diedrich, Jörn Dengjel, E. Wolfgang Kuehn, Anne Imberty, Winfried Römer

**Affiliations:** a Faculty of Biology, Albert-Ludwigs-University Freiburg, Freiburg, Germany; b Signalling Research Centres BIOSS and CIBSS, Albert-Ludwigs-University Freiburg, Freiburg, Germany; c Leibniz Institute for Experimental Virology (HPI), Hamburg, Germany; d Advanced Light and Fluorescence Microscopy Facility, Centre for Structural Systems Biology (CSSB), Hamburg, Germany; e Renal Division, Department of Medicine, Faculty of Medicine, Albert-Ludwigs-University Freiburg, Freiburg, Germany; f Department of Biology, University of Fribourg, Fribourg, Switzerland; g Department of Dermatology, Medical Center, Albert-Ludwigs-University Freiburg, Freiburg, Germany; h Université Grenoble Alpes, CNRS, CERMAV, Grenoble, France; i Freiburg Institute for Advanced Studies (FRIAS), Albert-Ludwigs-University Freiburg, Freiburg, Germany; University of California, Berkeley; Fred Hutchinson Cancer Research Center

**Keywords:** actin, epithelial cells, fucose, host cell invasion, lectin, primary cilium

## Abstract

The opportunistic bacterium Pseudomonas aeruginosa can infect mucosal tissues of the human body. To persist at the mucosal barrier, this highly adaptable pathogen has evolved many strategies, including invasion of host cells. Here, we show that the P. aeruginosa lectin LecB binds and cross-links fucosylated receptors at the apical plasma membrane of epithelial cells. This triggers a signaling cascade via Src kinases and phosphoinositide 3-kinase (PI3K), leading to the formation of patches enriched with the basolateral marker phosphatidylinositol (3,4,5)-trisphosphate (PIP_3_) at the apical plasma membrane. This identifies LecB as a causative bacterial factor for activating this well-known host cell response that is elicited upon apical binding of P. aeruginosa. Downstream from PI3K, Rac1 is activated to cause actin rearrangement and the outgrowth of protrusions at the apical plasma membrane. LecB-triggered PI3K activation also results in aberrant recruitment of caveolin-1 to the apical domain. In addition, we reveal a positive feedback loop between PI3K activation and apical caveolin-1 recruitment, which provides a mechanistic explanation for the previously observed implication of caveolin-1 in P. aeruginosa host cell invasion. Interestingly, LecB treatment also reversibly removes primary cilia. To directly prove the role of LecB for bacterial uptake, we coated bacterium-sized beads with LecB, which drastically enhanced their endocytosis. Furthermore, LecB deletion and LecB inhibition with l-fucose diminished the invasion efficiency of P. aeruginosa bacteria. Taken together, the results of our study identify LecB as a missing link that can explain how PI3K signaling and caveolin-1 recruitment are triggered to facilitate invasion of epithelial cells from the apical side by P. aeruginosa.

## INTRODUCTION

Pseudomonas aeruginosa is a ubiquitous environmental bacterium. Due to its intrinsic adaptability and the rise of multidrug-resistant strains, this bacterium poses a dangerous threat, especially in hospital settings. Accordingly, carbapenem-resistant P. aeruginosa strains were categorized by the World Health Organization (WHO) as priority 1 pathogens for which new antibiotics are critically required (1).

When infecting a human host, P. aeruginosa can switch between many lifestyles, including planktonic behavior and biofilm formation. In addition, evidence has accumulated during recent years that P. aeruginosa can also invade host cells. It has been demonstrated that P. aeruginosa is able to enter and survive (2, 3), move (4), and proliferate (5) in nonphagocytic cells. Moreover, after being taken up by macrophages, P. aeruginosa can escape phagosomes and eventually lyse the macrophages from the inside (6). The importance of the intracellular lifestyle for P. aeruginosa is supported by the observation that this bacterium has a whole arsenal of mechanisms to facilitate uptake by host cells. P. aeruginosa can invade by binding the remains of dead cells that are then taken up by surrounding cells through efferocytosis (7), by deploying the effector VgrG2b via its type VI secretion system (T6SS) to promote microtubule-dependent uptake (8), by utilizing cystic fibrosis transmembrane conductance regulator (CFTR) to stimulate caveolin-1-dependent endocytosis (9), and by interaction between the P. aeruginosa lectin LecA and the host cell glycosphingolipid globotriaosylceramide (Gb3) to facilitate invasion through a lipid zipper mechanism (10).

After incorporation by a human host, P. aeruginosa will typically interact with the apical plasma membranes of epithelial cells lining the mucosae. Interestingly, P. aeruginosa has developed mechanisms to manipulate the apical identity of these membranes. The hallmark of this process is the activation of phosphatidylinositol 3-kinase (PI3K), resulting in abnormal accumulation of phosphatidylinositol (3,4,5)-trisphosphate (PIP_3_) at the apical plasma membrane, which eventually generates patches with basolateral characteristics at the apical plasma membrane (11). This inversion of polarity has been suggested to help in the binding of P. aeruginosa to cells, since this bacterium uses different mechanisms to bind apical and basolateral plasma membranes (12). It is also crucial for host cell invasion by P. aeruginosa, because inhibition of PI3K signaling markedly reduces bacterial uptake (13). However, the exact mechanism by which P. aeruginosa is able to convert apical to basolateral plasma membrane is not clear. The formation of patches with basolateral characteristics at the apical plasma membrane requires the type III secretion system (T3SS) but, strikingly, does not require any of the toxins that are secreted via the T3SS (14, 15). To explain these observations, two hypotheses were suggested: host cell membrane damage through bacteria might be the initial event leading to basolateral patch formation, or PI3K signaling might be triggered by a still-unknown factor from P. aeruginosa (11).

Here, we provide data showing that the tetrameric fucose-specific lectin LecB (16), which is exposed at the outer membrane of P. aeruginosa (17, 18), represents the missing link. We showed already in a previous publication that purified LecB is able to bind receptors at the apical and basolateral plasma membrane of polarized Madin-Darby canine kidney (MDCK) cells (19). On the basolateral side, LecB was able to bind integrins, which led to integrin internalization and loss of epithelial polarity. Since only minute amounts of integrins are found at the apical side of polarized MDCK cells (19, 20), LecB did not dissolve epithelial polarity when applied only to the apical side (19). Here, we reveal that binding of LecB to fucosylated apical receptors on epithelial host cells was sufficient to trigger a different signaling cascade in order to promote cellular uptake of P. aeruginosa. Apical LecB binding led to Src signaling, followed by local PI3K activation, PIP_3_ patch formation at the apical plasma membrane, Rac1 signaling, and actin rearrangement to trigger the formation of protrusions in order to enable host cell invasion of P. aeruginosa. In addition, we show that caveolin-1 is recruited abnormally to apical membranes after LecB stimulation and that PI3K activation requires caveolin-1. These data suggest LecB as a unifying factor that facilitates and modulates many of the invasion mechanisms that have been reported for P. aeruginosa.

## RESULTS

### Apical LecB treatment triggers Src-PI3K/Akt signaling.

**FIG 1:**
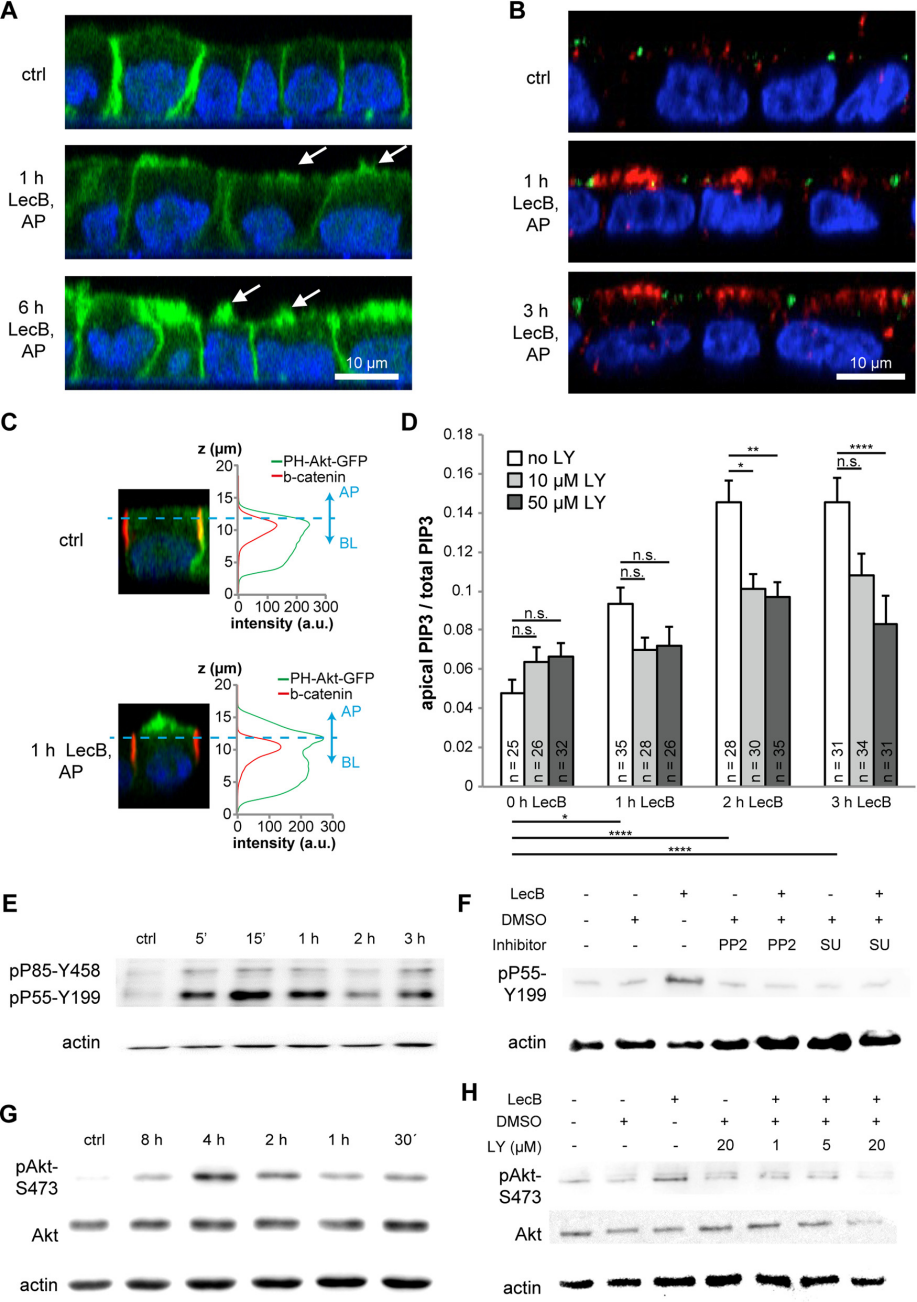
After binding to the apical plasma membrane of MDCK cells, LecB triggers an Src-PI3K/Akt signaling cascade. (A) MDCK cells stably expressing the PIP_3_ marker PH-Akt-GFP (green) were left untreated (ctrl) or treated from the apical (AP) side with LecB for the indicated time periods; nuclei were stained with DAPI (blue). White arrows point to apical protrusions resulting from LecB treatment. (B) MDCK cells were left untreated (ctrl) or treated with LecB as indicated, fixed, and then stained for active PI3K (pP85-Y458 and pP55-Y199; red) and ZO-1 (green); nuclei were stained with DAPI (blue). (C) MDCK cells stably expressing the PIP_3_ marker PH-Akt-GFP were treated with LecB from the apical (AP) side, fixed, and stained with β-catenin. To distinguish the apical and basolateral portion of the PH-Akt-GFP signal, β-catenin staining was utilized as a ruler. For the experiment, PH-Akt-GFP-positive cells were mixed with wt cells before seeding in a ratio of 1:10. This enabled an unbiased quantification by measuring the signals only from PH-Akt-GFP-positive cells that were surrounded by wt cells. a.u., arbitrary units. (D) Quantification of the results of the experiment described in the legend to panel C. The numbers indicated at the bottom of each bar represent the number of individual cells that were measured for each condition. Whereas cells treated with LecB show a time-dependent increase of the apical-to-total PH-Akt-GFP/PIP_3_ signal ratio, treatment with LY294002 (LY) reversed this effect. (E) MDCK cells were treated apically with LecB for the indicated times and subjected to Western blotting (WB) using an antibody recognizing active PI3K (pP85-Y458 and pP55-Y199). (F) MDCK cells were treated apically with LecB and PP2 (10 μM) or SU6656 (10 μM) for 1 h and subjected to WB utilizing an antibody recognizing active PI3K (pP55-Y199). DMSO, dimethyl sulfoxide. (G) MDCK cells were treated apically with LecB for the indicated times and subjected to WB utilizing an antibody recognizing active Akt (pAkt-S473). (H) MDCK cells were treated apically with LecB and indicated concentrations of LY294002 (LY) for 1 h and subjected to WB utilizing an antibody recognizing active Akt (pAkt-S473).

To more closely analyze the effects caused by the application of purified LecB to the apical side of polarized MDCK cells, we used MDCK cells stably expressing the green fluorescent protein (GFP)-tagged reporter PH-Akt-GFP, which indicates the localization of the lipid PIP_3_ (Fig. 1A) (21). In unstimulated cells, PH-Akt-GFP localized mainly to the basolateral plasma membrane, as expected from the role of PIP_3_ as a basolateral marker in polarized epithelial cells (21). In cells treated apically with LecB, PIP_3_ accumulated at the apical side and protrusions formed that were positive for PH-Akt-GFP (Fig. 1A, white arrows). This replicates the effects that were previously observed after interaction of whole P. aeruginosa bacteria with the apical plasma membrane of MDCK cells (22).

Importantly, we demonstrated already that apical LecB application did not disturb the integrity of tight junctions (19). Thus, LecB-mediated apical PIP_3_ accumulation cannot be explained by a loss of the barrier function of tight junctions. We therefore investigated whether activation of PI3K is the cause of apical PIP3 accumulation. Staining cells with antibodies recognizing active PI3K (pP85-Y458 and pP55-Y199) (23, 24) revealed a clearly visible recruitment and activation of PI3K to subapical regions in LecB-treated cells (Fig. 1B). In addition, incubating the cells with the broad-spectrum PI3K inhibitor LY294002 blocked the apical appearance of PH-Akt-GFP after LecB treatment (Fig. 1C and D). Activation of PI3K was also detectable by Western blotting (WB) and peaked at approximately 15 min after initiation of LecB stimulation (Fig. 1E). Upstream from PI3K, the activation of Src kinases was required, as demonstrated by the ability of the Src kinase inhibitors PP2 and SU6656 to block LecB-induced PI3K activation (Fig. 1F). LecB also activated Akt, for which phosphorylation at S473 was detectable after 30 min of LecB application and peaked at approximately 4 h (Fig. 1G). Akt signaling occurred downstream from PI3K, because the broad-spectrum PI3K inhibitor LY294002 blocked Akt activation (Fig. 1H). Further tests revealed that the PI3K subunit p110α was mainly responsible for LecB-mediated Akt activation, since the p110α-specific inhibitor PIK-75 blocked Akt activation (Fig. S1A in the supplemental material), whereas the p110β-specific inhibitor TGX-221 did not (Fig. S1B). Another fucose-binding lectin, Ulex europaeus agglutinin I (UEA-I), failed to replicate LecB-triggered Akt signaling (Fig. S1C), thus indicating that the observed effects are specific for LecB.

10.1128/mbio.00819-22.1 FIG S1 Control experiments related to the experiment whose results are shown in Fig. 1. (A) MDCK cells were treated apically with LecB and with the indicated concentrations of the p110α-specific inhibitor PIK-75 for 1 h, and Akt activation (pAkt-S473) was probed by Western blotting (WB). (B) MDCK cells were treated apically with LecB and with the indicated concentrations of the p110β-specific inhibitor TGX-221 for 1 h, and Akt activation (pAkt-S473) was probed by WB. (C) MDCK cells were treated apically with 50 μg/mL UEA-I or LecB, and Akt activation (pAkt-S473) was probed by WB. Download FIG S1, DOCX file, 0.07 MB. Copyright © 2022 Thuenauer et al. 2022 Thuenauer et al. https://creativecommons.org/licenses/by/4.0/ This content is distributed under the terms of the Creative Commons Attribution 4.0 International license.

To demonstrate that LecB-mediated PI3K/Akt activation is not limited to MDCK cells, we carried out experiments in other cell lines. We chose H1975 lung epithelial cells because P. aeruginosa frequently infects lungs. Whereas MDCK cells are Gb3-negative, H1975 cells express Gb3 (Fig. S2). The glycosphingolipid Gb3 has been previously found to be required for LecA-mediated internalization of P. aeruginosa (10). In H1975 cells, LecB also triggered Akt activation, in a dose- and time-dependent manner (Fig. S3A to D) and dependent on PI3K (Fig. S3E and F, showing the results of experiments using the pan-PI3K inhibitors wortmannin and LY294002 and the Akt inhibitor triciribine). As a further control, we verified that soluble l-fucose, which prevents LecB from engaging with host cell receptors, is able to inhibit LecB-triggered Akt signaling (Fig. S3G and H). This demonstrates that LecB binding to fucosylated receptors is necessary to trigger PI3K/Akt signaling and also validates the purity of our LecB preparation.

10.1128/mbio.00819-22.2 FIG S2 Evaluation of Gb3 expression in MDCK cells and H1975 cells. MDCK cells (A) and H1975 cells (B) were seeded sparsely on glass cover slips and then incubated for 30 min at 37°C with 1 μg/mL StxB-Alexa Fluor 488 (green). StxB is a lectin that specifically binds the glycosphingolipid Gb3. After fixation, cell nuclei were stained with DAPI (blue) and all samples were imaged with a confocal microscope using the same settings to ensure comparability. For each cell type, three different randomly chosen regions of interest (ROI) are displayed. Whereas MDCK cells do not bind StxB and are therefore Gb3 negative, H1975 cells show detectable binding of StxB and are hence expressing Gb3. Download FIG S2, DOCX file, 0.2 MB. Copyright © 2022 Thuenauer et al. 2022 Thuenauer et al. https://creativecommons.org/licenses/by/4.0/ This content is distributed under the terms of the Creative Commons Attribution 4.0 International license.

10.1128/mbio.00819-22.3 FIG S3 In H1975 lung epithelial cells, LecB also activates PI3K/Akt signaling. (A to C) H1975 cells were treated with LecB as indicated, and Akt activation (pAkt-S473) was probed by WB. The image depicts a representative Western blot; quantifications from *n* = 3 independent experiments for the dose dependence and the time dependence are depicted in panels B and C, respectively. (D) H1975 cells were treated with LecB-Cy3 (red) and fixed, and activated Akt was visualized by an antibody specific for pAkt-S473 (green). (E and F) H1975 cells were treated with LecB and PI3K inhibitors wortmannin (100 nM), LY294002 (10 μM), and the Akt inhibitor triciribine (10 μM) for 1 h, and Akt activation (pAkt-S473) was probed by WB. (E) Representative Western blot. (F) Quantification of results from *n* = 3 independent experiments. (G and H) H1975 cells were treated with LecB and l-fucose (43 mM) for 1 h, and Akt activation (pAkt-S473) was probed by WB. (G) Representative Western blot. (H) Quantification of the results from *n* = 3 independent experiments. Download FIG S3, DOCX file, 0.3 MB. Copyright © 2022 Thuenauer et al. 2022 Thuenauer et al. https://creativecommons.org/licenses/by/4.0/ This content is distributed under the terms of the Creative Commons Attribution 4.0 International license.

To identify apical interaction partners of LecB, we applied LecB-biotin apically to polarized MDCK cells, lysed them, and precipitated LecB-receptor complexes with streptavidin beads. Mass spectrometry (MS) analysis revealed 12 profoundly enriched proteins (Table S1), underscoring the property of LecB of binding to multiple receptors. However, this property also prevented us from singling out a receptor that was responsible for LecB-triggered PI3K signaling, since the list included several proteins for which a capacity to elicit PI3K signaling was known (CEACAM1 [25, 26], mucin-1 [27], ICAM1 [28], and podocalyxin [29, 30]).

10.1128/mbio.00819-22.8 TABLE S1 List of apical LecB interaction partners identified by SILAC MS. Download Table S1, DOCX file, 0.01 MB. Copyright © 2022 Thuenauer et al. 2022 Thuenauer et al. https://creativecommons.org/licenses/by/4.0/ This content is distributed under the terms of the Creative Commons Attribution 4.0 International license.

Taken together, these findings show that after binding to fucosylated receptors at the plasma membrane of epithelial cells, LecB triggered an Src-PI3K/Akt signaling cascade, which replicated the cellular responses that were observed after binding of live P. aeruginosa cells to apical membranes (13).

### Coating beads with LecB and expression of LecB by P. aeruginosa both enhance their apical uptake.

**FIG 2:**
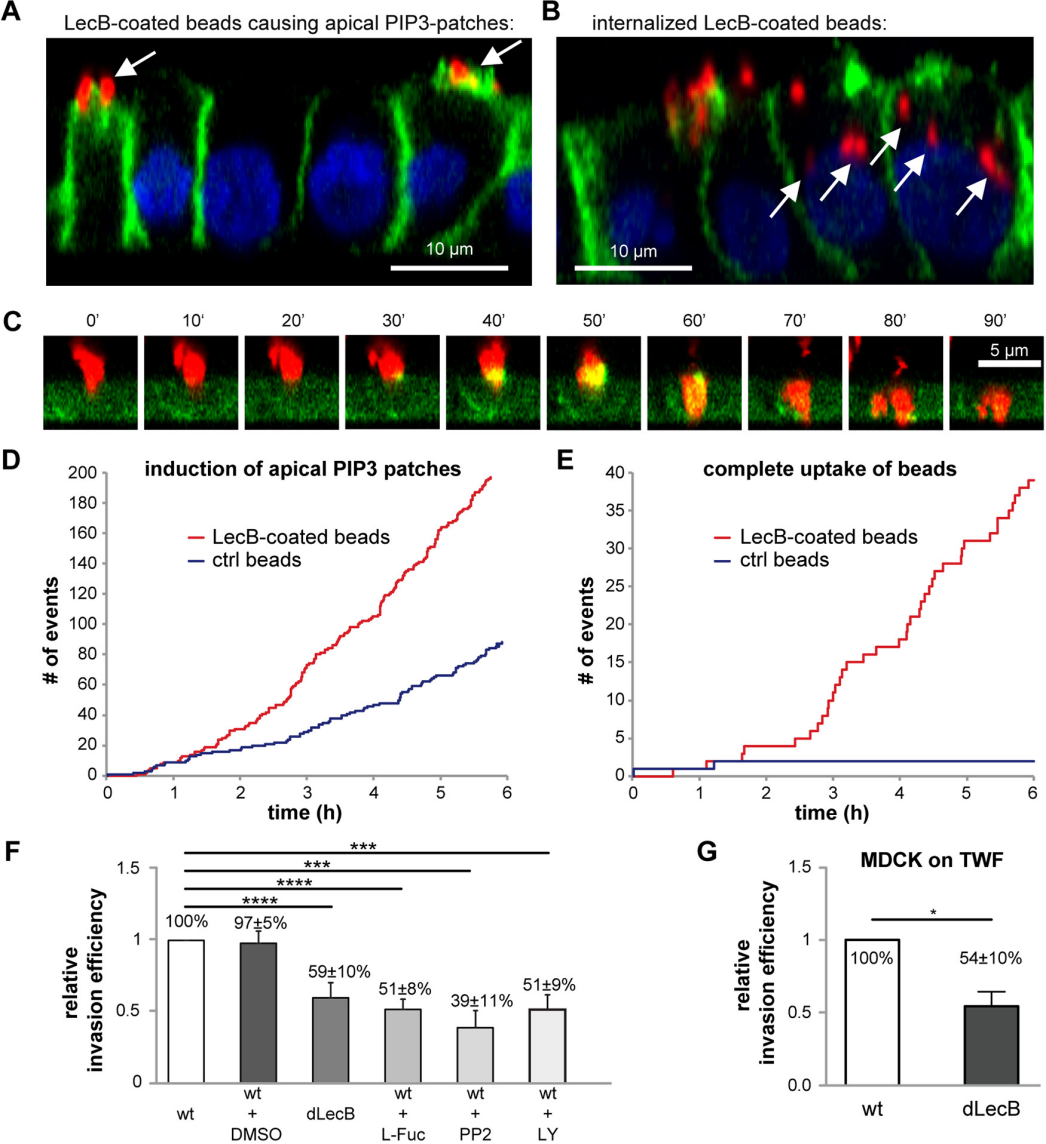
LecB facilitates apical uptake of beads and apical invasion of P. aeruginosa. (A and B) Red fluorescent LecB-coated bacterium-sized beads with 1-μm diameter were apically applied to MDCK cells stably expressing the PIP_3_ marker PH-Akt-GFP (green) for 6 h; nuclei were stained with DAPI (blue). (A) Instances of beads causing apical PIP_3_ patches (white arrows). (B) Fully internalized beads are depicted (white arrows). (C to E) MDCK cells stably expressing PH-Akt-GFP (green) were allowed to polarize on cover glasses. Red fluorescent beads of 1-μm diameter coated with LecB were applied, and live-cell confocal imaging was performed. The images show apicobasal cross sections extracted from confocal image stacks. (D and E) The number of induced apical PIP_3_-patches (D) and the number of beads that are completely taken up over time (E) are depicted for biotin-coated beads (ctrl) and LecB-coated beads. (F) Using an amikacin protection assay, the invasion efficiencies of wild-type (wt) and LecB-deficient (dLecB) PAO1 applied at an MOI of 50 for 2 h on the apical side of polarized MDCK cells grown in 24-well plates were determined. In addition, the invasion efficiencies for bacteria preincubated with 100 mg/mL l-fucose (L-Fuc) and for cells treated with PP2 (10 μM) and LY294002 (LY; 10 μM) were measured. Mean values and SEM from *n* = 8 experiments are shown. (G) Amikacin protection assays measuring the apical invasion of PAO1-wt and PAO1-dLecB in MDCK cells grown on transwell filters. Invasion for 2 h, MOI = 50, *n* = 3.

To more realistically model the geometry during infection with P. aeruginosa, we utilized bacterium-sized beads that were coated with LecB. In pilot experiments using cell fixation, LecB-coated beads were seen to bind to the apical plasma membrane of polarized MDCK cells and to cause local apical accumulation of PH-Akt-GFP/PIP_3_ (Fig. 2A), and many beads were found to be completely internalized by cells (Fig. 2B). Live-cell microscopy experiments revealed that apical PH-Akt-GFP/PIP_3_ accumulation is a transient event that occurs before apical uptake of beads by MDCK cells (Fig. 2C, Movie S1). Detailed quantification showed that biotin-coated control beads were able to trigger apical PH-Akt-GFP/PIP_3_ bursts to some extent, but at a much lower rate than LecB-coated beads (Fig. 2D). Interestingly, the PH-Akt-GFP/PIP_3_ bursts caused by control beads were hardly sufficient to mediate cellular uptake, whereas the LecB-coated beads were taken up extensively (Fig. 2E). In addition, LecB treatment stimulated macropinocytotic uptake of dextran in H1975 cells (Fig. S4A and B), which provides further evidence that LecB activates cellular uptake mechanisms.

10.1128/mbio.00819-22.4 FIG S4 LecB stimulates macropinocytosis and facilitates P. aeruginosa invasion in H1975 cells. (A and B) Representative images of untreated H1975 cells and cells treated with LecB-Cy3 for 2 h (red). To probe macropinocytosis, cells were also incubated with 0.2 μM FITC-dextran (green); nuclei were stained with DAPI (blue). White arrows point to internalized FITC-dextran colocalizing with LecB-Cy3. (B) Quantification of FITC-dextran uptake in untreated and LecB-treated cells; *n* = 3. (C and D) Amikacin protection assays measuring the invasion of PAO1-wt and PAO1-dLecB (C) and PAO1-wt preincubated with 100 mg/mL l-fucose (D) in H1975 cells. Invasion for 2 h, MOI = 50, *n* = 3. Download FIG S4, DOCX file, 0.2 MB. Copyright © 2022 Thuenauer et al. 2022 Thuenauer et al. https://creativecommons.org/licenses/by/4.0/ This content is distributed under the terms of the Creative Commons Attribution 4.0 International license.

10.1128/mbio.00819-22.10 MOVIE S1 Time-lapse movie of the internalization of a LecB-coated bead cluster (red) into polarized MDCK cells expressing PH-Akt-GFP (green). The time between individual frames is 10 min, and the movie is replayed at 3 frames per second. Download Movie S1, AVI file, 0.04 MB. Copyright © 2022 Thuenauer et al. 2022 Thuenauer et al. https://creativecommons.org/licenses/by/4.0/ This content is distributed under the terms of the Creative Commons Attribution 4.0 International license.

Motivated by these results, we investigated whether the expression of LecB influences host cell uptake of live P. aeruginosa bacteria. Indeed, abrogation of LecB expression in P. aeruginosa (dLecB) and blockage of LecB with l-fucose diminished the apical uptake of P. aeruginosa in polarized MDCK cells (Fig. 2F). In accordance with previous studies (13, 31), inhibition of Src kinases with PP2 and inhibition of PI3K with LY294002 also decreased P. aeruginosa uptake (Fig. 2F). Due to the easier handling, the experiments whose results are shown in Fig. 2F were carried out with MDCK cells grown in 24-well plates. For verification, we repeated them with transwell filter-grown MDCK cells, which yielded comparable results (Fig. 2G). Of note, the association of wild-type (wt) and dLecB P. aeruginosa with polarized MDCK cells was not significantly different (Fig. S5), which suggests that the observed decrease of invasion efficiency upon deletion of LecB was due to LecB-mediated signaling and not due to reduced host cell binding. In H1975 cells, the uptake of P. aeruginosa was also lowered by LecB deletion (Fig. S4C) and l-fucose treatment (Fig. S4D).

10.1128/mbio.00819-22.5 FIG S5 Comparison of cell association of wt and dLecB P. aeruginosa. Wild-type and dLecB P. aeruginosa cells were applied to polarized MDCK cells at an MOI of 50 for 2 h. Afterwards, cells were lysed with 0.25% (vol/vol) Triton X-100. Serial dilutions of the cell extracts were made and plated on LB-Miller agar plates containing gentamicin (60 μg/mL) for counting. This corresponds to the procedure for determining the total number of bacteria in the amikacin protection assays. The graph shows the mean values of counted bacteria from *n* = 8 experiments. Download FIG S5, DOCX file, 0.04 MB. Copyright © 2022 Thuenauer et al. 2022 Thuenauer et al. https://creativecommons.org/licenses/by/4.0/ This content is distributed under the terms of the Creative Commons Attribution 4.0 International license.

Taken together, these data demonstrate that LecB promotes the uptake of P. aeruginosa from the apical side in polarized epithelial cells.

### LecB-mediated PI3K signaling leads to Rac activation and actin rearrangement.

**FIG 3:**
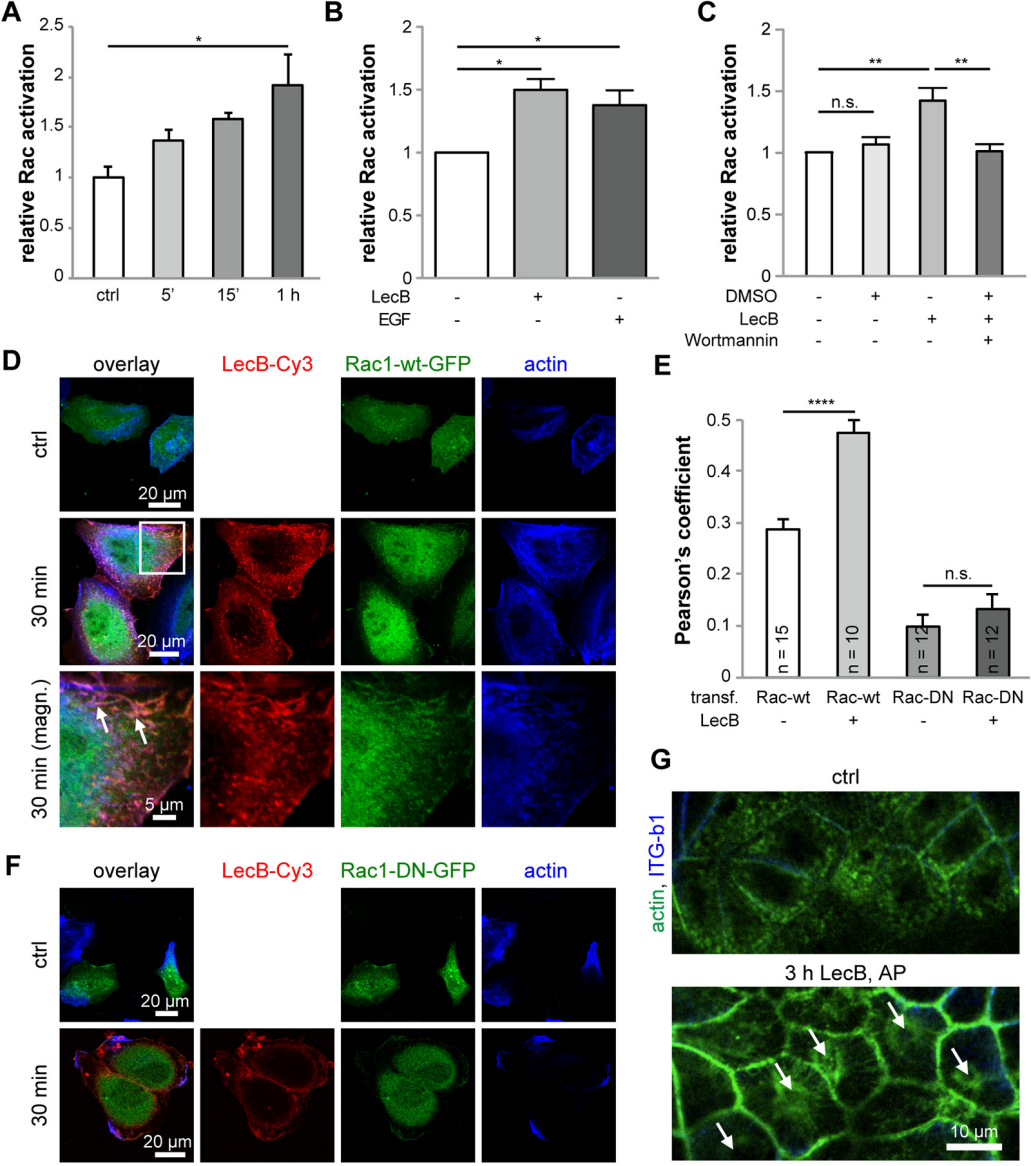
Apical LecB stimulation leads to Rac activation and actin rearrangement. (A) The activation of Rac upon apical LecB treatment of MDCK cells was measured using a Rac123-G-LISA assay; *n* = 3. (B) H1975 cells were treated with LecB or EGF (20 nM), and Rac activation was measured using a Rac123-G-LISA assay; *n* = 3. (C) H1975 cells were treated with LecB and wortmannin (100 nM), and Rac activation was measured using a Rac123-G-LISA assay; *n* = 6. (D to F) H1975 cells transfected with Rac1-wt-GFP (green) (D) or Rac1-DN-GFP (green) (F) were treated with LecB-Cy3 (red) as indicated, fixed, and stained for actin with phalloidin-Atto 647 (blue). (D) White arrows point to ruffle-like structures where LecB, Rac1-wt-GFP, and actin colocalized. (E) The Pearson’s colocalization coefficient between Rac1-wt-GFP or Rac1-DN-GFP and actin in cells untreated or treated with LecB-Cy3 was determined in individual cells, and the average was calculated. (G) MDCK cells treated with LecB as indicated were fixed and stained with phalloidin-Atto 488 to stain actin (green) and β1-integrin (blue). Lateral confocal cross sections along the apical poles of the cells are displayed.

To better understand the cellular response upon apical LecB stimulation, we investigated how PI3K activation is linked to P. aeruginosa uptake. Motivated by the known correlations between PI3K and Rac activation (32) and the reported implication of Rac in P. aeruginosa internalization (31), we carried out experiments using Rac123-G-LISA assays to test the capability of LecB to activate Rac. We found that apically applied LecB activated Rac in a time-dependent manner in MDCK cells (Fig. 3A) and also in H1975 cells (Fig. 3B). The PI3K inhibitor wortmannin blocked LecB-mediated Rac activation (Fig. 3C), indicating that PI3K activation occurred upstream from Rac activation.

To investigate the consequences of LecB-mediated Rac activation on the actin cytoskeleton further, we utilized unpolarized H1975. The reason for this is that this allowed us to use overexpression of dominant-negative (DN) Rac1, which would result in unwanted side effects in polarized MDCK cells, because Rac1 also has roles during the polarization of MDCK cells (33). In sparsely seeded H1975 cells, LecB caused ruffle-like structures (Fig. 3D), and LecB colocalized with transfected Rac1-wt-GFP and actin in the ruffle-like regions (Fig. 3D, white arrows). To verify that LecB induced recruitment of Rac1-wt-GFP toward actin, we determined the Pearson’s colocalization coefficient between Rac1-wt-GFP and actin, which increased significantly in LecB-treated cells (Fig. 3E). This was not the case when DN Rac1-GFP (Rac1-DN-GFP) was overexpressed in H1975 cells (Fig. 3F and E), showing the requirement of functional Rac for this effect. For verification, we repeated the experiment in untransfected H1975 cells using antibodies recognizing endogenous Rac1 (Fig. S6). Consistently, recruitment of Rac to actin upon LecB stimulation occurred as well in this experiment.

10.1128/mbio.00819-22.6 FIG S6 Control experiment related to the experiment whose results are shown in Fig. 3. (A and B) H1975 cells were treated with LecB-Cy3 (red) as indicated, fixed, stained for actin with phalloidin-Atto 647 (blue) and endogenous Rac1 (green), and then imaged with a confocal microscope. (A) Representative images. (B) The Pearson’s colocalization coefficient between endogenous Rac1 and actin was determined in individual cells, and the average was calculated. Download FIG S6, DOCX file, 0.1 MB. Copyright © 2022 Thuenauer et al. 2022 Thuenauer et al. https://creativecommons.org/licenses/by/4.0/ This content is distributed under the terms of the Creative Commons Attribution 4.0 International license.

Apical application of LecB also led to substantial rearrangement of actin at the apical cell pole of MDCK cells (Fig. 3G). In untreated cells, dotted structures representing microvilli and the central actin-devoid region of the periciliary membrane and the primary cilium (34–36) were visible. In cells treated apically for 3 h with LecB, this subapical organization of the actin cytoskeleton was completely lost. Actin was recruited to lateral aspects of the cell membrane, and actin stress fibers constricting around the central position of the outgrowth of the primary cilium (Fig. 3G, white arrows) appeared.

In summary, the results of these experiments show that LecB-triggered PI3K signaling leads to Rac activation and actin rearrangement. All these processes have been previously observed during internalization of P. aeruginosa (22, 31, 37), thus further underscoring the role of LecB for P. aeruginosa host cell invasion.

### Apical LecB treatment reversibly removes primary cilia.

**FIG 4:**
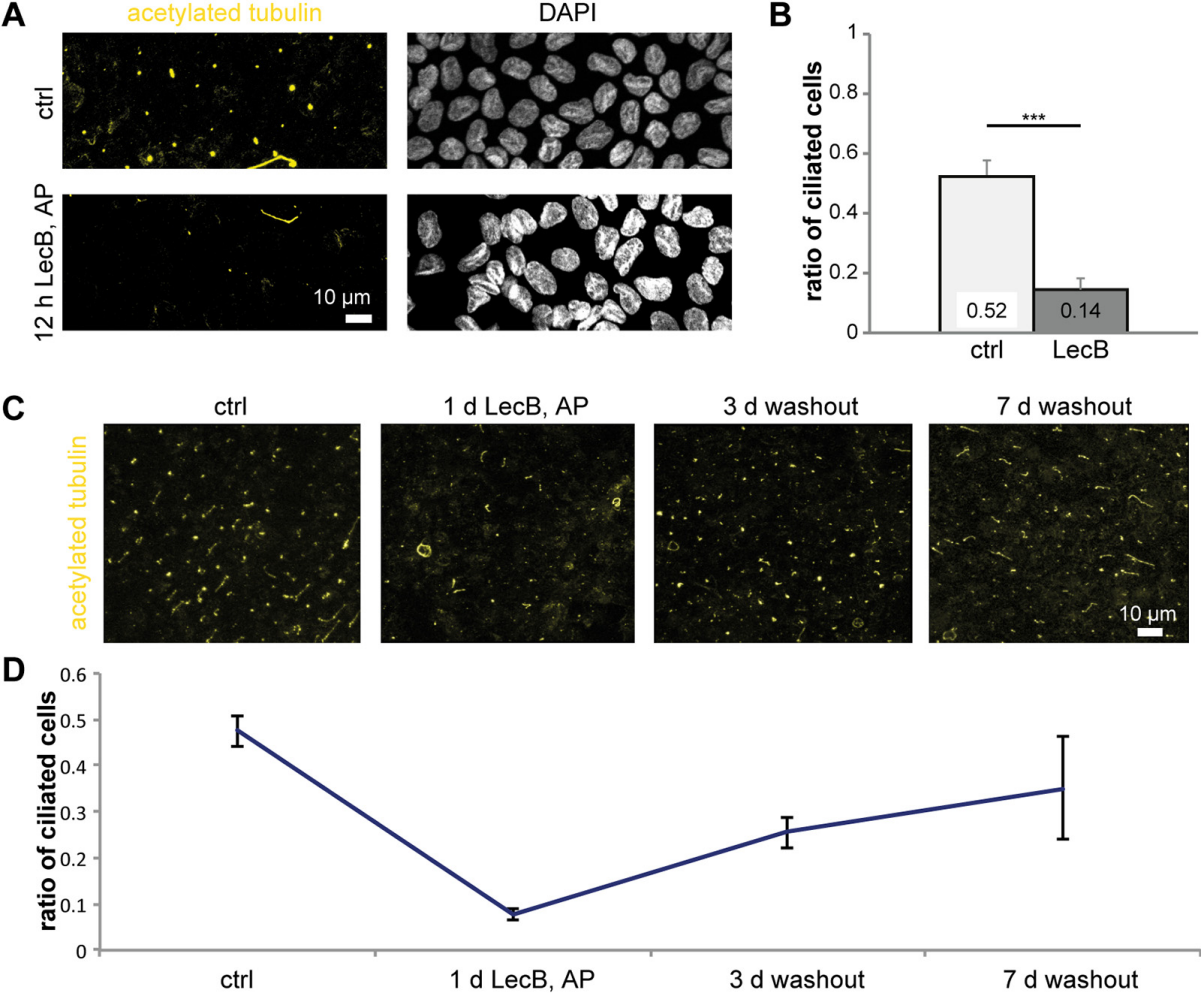
Apical treatment with LecB removes primary cilia in a reversible manner. (A to D) MDCK cells were grown on glass coverslips for 10 days. After the indicated treatments, cells were fixed, and immunofluorescence staining was performed for acetylated tubulin (yellow) to visualize primary cilia. (A) Nuclei were additionally stained with DAPI (white). Maximum intensity projections of confocal image stacks covering total cell heights are shown. (B) The ratio of ciliated cells was calculated by dividing the number of visible cilia by the total number of cells. Five fields of view (125 μm by 125 μm) were summed up for *n* = 1, and the results from *n* = 3 independent experiments were averaged. (C) MDCK cells were treated with LecB, followed by washout as indicated. (D) Quantification of the results of the experiment shown in panel C.

Motivated by our observation that LecB treatment led to the formation of actin stress fibers that appeared to constrict around the basis of the primary cilium, we investigated the effects of LecB on the primary cilium. Interestingly, apical application of LecB removed primary cilia from polarized MDCK cells (Fig. 4A and B) within 12 h. This effect was reversible after washout of LecB (Fig. 4C and D). Although the potential physiological consequences of loss of primary cilia during P. aeruginosa infection remain to be investigated, this finding underscores the massive extent of LecB-mediated actin rearrangement.

### LecB triggers a feedback loop between caveolin-1 recruitment and PI3K activation.

**FIG 5:**
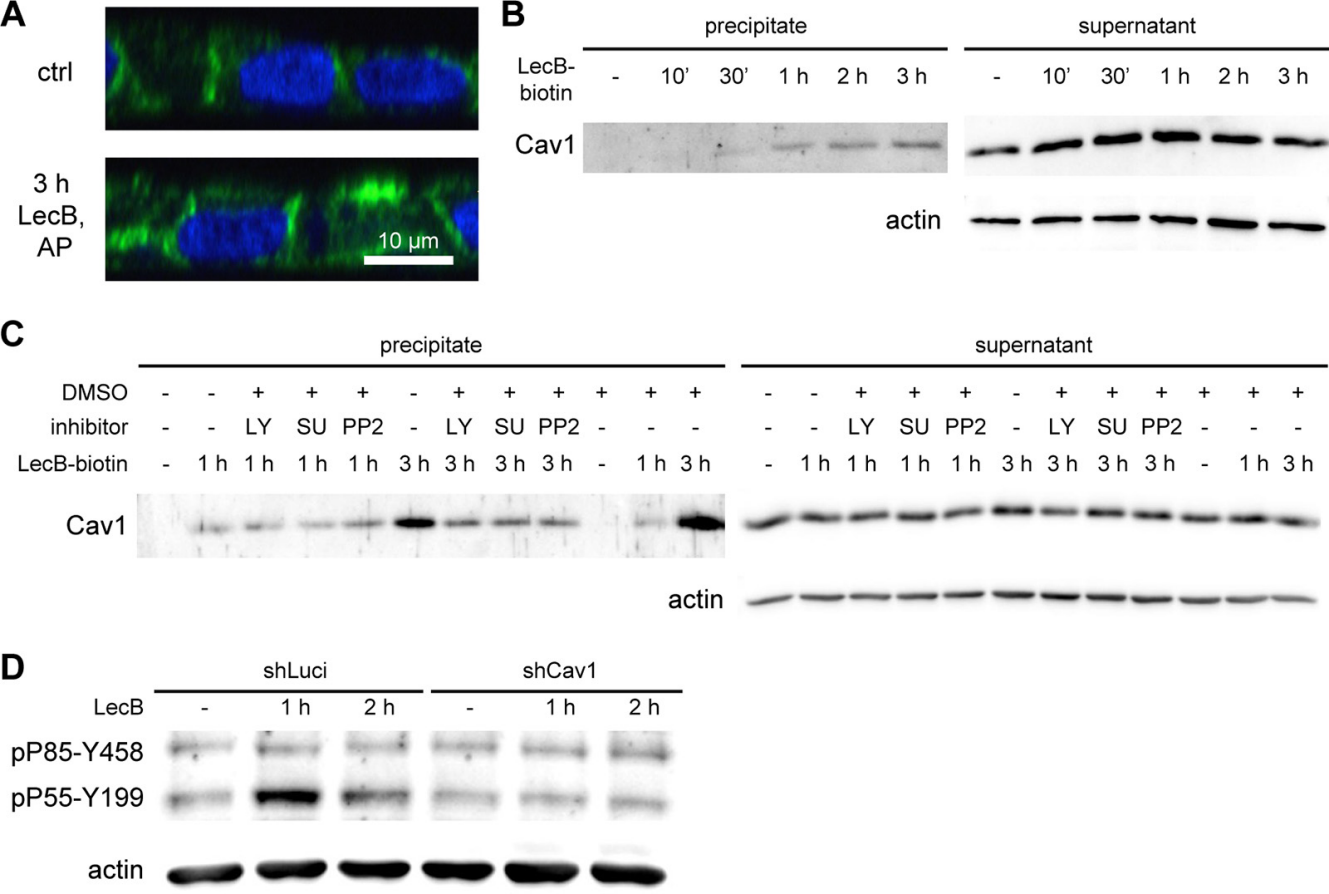
Caveolin-1 is essential for LecB-triggered PI3K signaling. (A) Polarized MDCK cells were treated apically (AP) with LecB as indicated, fixed, and stained for caveolin-1 (green); nuclei were stained with DAPI (blue). (B and C) LecB-biotin was apically applied to polarized MDCK cells for the indicated times. After cell lysis, LecB-biotin-receptor complexes were precipitated with streptavidin beads, and the precipitate and the supernatant were probed by WB for caveolin-1. (C) Cells were additionally treated with LY294002 (LY; 10 μM), PP2 (10 μM), or SU6656 (SU; 10 μM). (D) Polarized MDCK cells expressing a control shRNA (shLuci) and caveolin-1 knockdown MDCK cells (shCav1) were treated apically with LecB as indicated and subjected to WB using an antibody recognizing active PI3K (pP85-Y458 and pP55-Y199).

Interestingly, we also found caveolin-1 in the MS screen of LecB interactors (Table S1). Since caveolin-1 is a cytosolic protein, it presumably coprecipitated with LecB-interacting receptors. Motivated by this finding, we further investigated the behavior of caveolin-1 after LecB treatment. In undisturbed MDCK cells, caveolin-1 preferentially localized to the basolateral plasma membrane, as observed before (Fig. 5A) (38). However, apical LecB treatment resulted in abnormal recruitment of caveolin-1 toward the apical cell pole (Fig. 5A). In addition, the recruitment of caveolin-1 to LecB-receptor complexes was verifiable by WB and increased in a time-dependent manner (Fig. 5B). Interestingly, blocking Src kinases with SU6656 or PP2 and blocking PI3K with LY294002 diminished the coprecipitation of caveolin-1 in complexes with LecB-biotin (Fig. 5C). To directly investigate the requirement of caveolin-1 for LecB-mediated PI3K activation, we knocked down caveolin-1 in MDCK cells using small hairpin RNA (shRNA) (Fig. S7). Caveolin-1 knockdown almost completely suppressed PI3K activation upon LecB treatment (Fig. 5D).

10.1128/mbio.00819-22.7 FIG S7 Verification of caveolin-1 knockdown in shCav1 cells. (A and B) The expression of caveolin-1 in caveolin-1 knockdown MDCK cells (shCav1) and wild-type MDCK cells (wt) was assessed by WB. (A) Representative Western blots. (B) Quantification of caveolin-1 knockdown from *n* = 3 independent experiments. To calculate the normalized caveolin-1 expression, the caveolin-1 band intensities were divided by actin band intensities for each individual sample and averaged. Error bars represent SEM, statistical significance was evaluated by a paired, two-sided *t* test, ** denotes *P* < 0.01. Download FIG S7, DOCX file, 0.05 MB. Copyright © 2022 Thuenauer et al. 2022 Thuenauer et al. https://creativecommons.org/licenses/by/4.0/ This content is distributed under the terms of the Creative Commons Attribution 4.0 International license.

Taken together, these data demonstrate that caveolin-1 is apically recruited by LecB stimulation and that this recruitment requires activation of Src kinases and PI3K, whereas caveolin-1 is also required for LecB-triggered PI3K activation. This constitutes a positive feedback loop between caveolin-1 recruitment and PI3K activation.

## DISCUSSION

Here, we demonstrate that LecB is able to trigger an Src-PI3K-Rac signaling cascade, which is modulated by caveolin-1 and leads to actin rearrangement and protrusion formation in order to promote cellular uptake of P. aeruginosa bacteria. This adds LecB-triggered signaling to the growing list of P. aeruginosa host cell invasion mechanisms, which provokes the question of why this bacterium has evolved so many invasion mechanisms and how LecB fits in.

The multitude of invasion mechanisms might be rooted in the adaptability of this opportunistic pathogen. P. aeruginosa can infect the respiratory tract, urinary tract, eye, and skin (39), and it was demonstrated that this bacterium can invade epithelial cells from the lung (9), cornea (2), and kidneys (22, 40). Considering this diversity, it makes sense that P. aeruginosa possesses many invasion mechanisms, which might be used by the bacterium depending on the type of host cell encountered. One example is lipid zipper-type invasion, which requires interaction between LecA from P. aeruginosa and the glycosphingolipid Gb3 as the host cell factor (10). However, this lipid is not expressed in all epithelial cell types. For example, the MDCK cells used in this study do not express Gb3 (Fig. S2) (41). Nevertheless, P. aeruginosa successfully invaded MDCK cells, and thus, it uses alternative pathways like LecB-mediated signaling, as we demonstrated here. In addition, we show that LecB deletion in P. aeruginosa also decreased the invasion efficiency in H1975 cells, which we identified as Gb3 positive (Fig. S2), and it has been demonstrated previously that Gb3 expression in MDCK cells increased the invasion efficiency (10). These examples suggest that invasion mechanisms, such as LecA- and LecB-dependent invasion, are not exclusive but rather function in an additive manner. Our data provide an additional line of evidence for a cooperative function of invasion mechanisms. Coating of bacterium-sized beads with LecB markedly stimulated their uptake into cells, thus demonstrating that LecB alone is sufficient for stimulating cellular uptake. But LecB deletion or blocking LecB with l-fucose did not decrease the internalization of P. aeruginosa bacteria to the same extent as inhibition of Src kinases and PI3K did. This hints at other bacterial factors that are also able to cause PI3K-dependent uptake into host cells. A potential candidate is type IV pili, since deletion of pili led to a small but significant reduction of PI3K/Akt activation upon apical application of P. aeruginosa to polarized Calu-3 cells (12).

How is LecB able to trigger the Src-PI3K-Rac-actin signaling cascade? By MS analysis, we showed that LecB binds multiple apical receptors capable of triggering PI3K-signaling: CEACAM1 (25, 26), Mucin-1 (27), ICAM1 (28), and podocalyxin (29, 30). This makes it on one hand more robust for the bacterium to trigger the desired response, but it also makes it difficult for us to isolate a detailed mechanistic picture of LecB action at the apical cell membrane. We hypothesize that LecB has, due to being a tetramer that offers four binding sites (42), the capacity to cross-link and cluster different receptors (19, 43), which is a general mechanism to activate receptor-mediated signaling cascades at the cell membrane. The data we present here provide two independent lines of evidence for this hypothesis. The first line derives from our control experiments with the lectin UEA-I. UEA-I is also able to bind fucose, but it has only two binding sites (44). This makes UEA-I a less ideal cross-linker than the tetrameric LecB, which was shown to be capable of cross-linking fucosylated lipids and integrins (19, 43). Consequently, we found that UEA-I was not capable of eliciting PI3K signaling. This confirms that binding to fucosylated receptors is not enough and additional cross-linking, as in the case of LecB, is required for triggering PI3K signaling. The second line of evidence can be deduced from our experiments regarding caveolin-1. It has been shown that receptor cross-linking is sufficient to aberrantly induce caveolin-1-containing caveolae at the apical plasma membrane of epithelial cells (38, 45). LecB application at the apical plasma membrane also caused the abnormal recruitment of caveolin-1 to the apical plasma membrane, which can be explained by assuming that LecB cross-linked receptors. In addition, the fact that caveolin-1 knockdown abrogated LecB-mediated PI3K activation, together with our finding that caveolin-1 recruitment could be blocked by PI3K inhibitors, suggests that there exists a positive feedback loop between PI3K activation and caveolin-1 recruitment. This is strongly supported by our observation that caveolin-1 coprecipitation with apical LecB receptors increased in a time-dependent manner. This also offers an explanation for the previously reported role of caveolin-1 for P. aeruginosa host cell invasion (9).

There has been speculation in the literature about the initial events that trigger the basolateral patch formation at the apical membrane by P. aeruginosa, and two possible hypotheses were offered (11): Either membrane damage could be responsible, or a still unknown bacterial factor causes the required PI3K activation. Our results favor the second hypothesis. Binding and cross-linking of apical receptors by LecB offer a direct explanation for PI3K activation and, thus, identify LecB as the unknown bacterial factor. In addition, we previously reported that application of purified LecB to the apical plasma membrane of MDCK cells does not induce membrane damage, as measured by trypan blue assays that use the fluorescence of trypan blue as a sensitive readout (19). Likewise, tight junction integrity was not affected by apical application of LecB (19). This is in agreement with the finding by others that the formation of PIP_3_-rich protrusions during infection with P. aeruginosa did not compromise tight junctions (11). This finding also excludes the possibility that LecB-triggered apical PIP_3_ accumulation occurred by diffusive spreading of PIP_3_ from the basolateral plasma membrane and additionally proves that apical PIP_3_ accumulation was due to LecB-mediated local PI3K activity at the apical plasma membrane.

The involvement of Rac1 for P. aeruginosa internalization through the LecB-triggered cascade we describe here will need further clarification. Specifically, the P. aeruginosa exotoxin S and exotoxin T are known to contain N-terminal RhoGTPase activating protein (RhoGAP) domains, which can hydrolyze GTP to GDP in Rho, Rac, and Cdc42, leading to cytoskeletal depolymerization and countering host cell invasion (46, 47). It will be interesting to investigate whether varying expression levels of LecB, exotoxin S, and exotoxin T cause more or less invasive behavior of P. aeruginosa.

In conclusion, our results identify LecB as a novel bacterial factor that promotes uptake of P. aeruginosa bacteria from the apical side of epithelial cells. Our data suggest that LecB represents a missing link that provides a unifying explanation for many observations that have been made during host cell invasion by P. aeruginosa. We revealed that LecB is sufficient to trigger the well-known Src-PI3K-Rac signaling cascade (11), which is required for basolateral patch formation at the apical plasma membrane and host cell invasion. LecB-mediated signaling also provides additional rationales for the previously found implication of caveolin-1 in P. aeruginosa invasion (9), since we identified here a LecB-triggered positive feedback loop between PI3K activation and caveolin-1 recruitment to the apical plasma membrane.

## MATERIALS AND METHODS

### Antibodies, plasmids, and reagents.

The antibodies used are listed in Table S2. The plasmid pPH-Akt-GFP encoding PH-Akt-GFP was a gift from Tamas Balla (Addgene plasmid no. 51465). The plasmids encoding wild-type Rac1 tagged with GFP (Rac1-wt-GFP) and a mutant protein bearing a change of T to N at position 17 (Rac1-T17N) and tagged with GFP (Rac1-DN-GFP) were kindly provided by Stefan Linder (University Hospital Hamburg-Eppendorf, Germany).

10.1128/mbio.00819-22.9 TABLE S2 Lists of primary and secondary antibodies used (WB, Western blot; IF, immunofluorescence). Download Table S2, DOCX file, 0.01 MB. Copyright © 2022 Thuenauer et al. 2022 Thuenauer et al. https://creativecommons.org/licenses/by/4.0/ This content is distributed under the terms of the Creative Commons Attribution 4.0 International license.

Recombinant LecB was produced in Escherichia coli BL21(DE3) cells and purified with affinity columns as previously described (19). LecB and fluorophore-conjugated LecB were used at a concentration of 50 μg/mL (4.3 μM) unless stated otherwise. The B-subunit of Shiga toxin 1 (StxB) recombinantly produced in Escherichia coli was from Sigma-Aldrich. LY294002, wortmannin, PP2, SU6656, PIK-75, TGX-221, and triciribine were from Selleckchem. UEA-I was from Vector Labs. Human epidermal growth factor (EGF), l-fucose (6-deoxy-l-galactose), and fluorescein isothiocyanate (FITC)-dextran (70 kDa) were from Sigma-Aldrich. Phalloidin-Atto 488 and phalloidin-Atto 647 were from Atto-Tec.

### Mammalian cell culture and creation of stable cell lines.

MDCK strain II cells were cultured in Dulbecco’s modified Eagle’s medium (DMEM) supplemented with 5% fetal calf serum (FCS) at 37°C and 5% CO_2_. H1975 cells were maintained in Roswell Park Memorial Institute (RPMI) 1640 medium supplemented with 10% FCS at 37°C and 5% CO_2_. For generating polarized MDCK monolayers, 3 × 10^5^ MDCK cells were seeded on transwell filters (12-well format, 0.4-μm pore size, polycarbonate membrane, product number 3401; Corning) and cultured for 4 days before experiments. For experiments with H1975 cells, 3 × 10^4^ cells were seeded per 12-mm glass cover slip placed in a 24-well plate and cultured for 1 day. For the creation of the MDCK cell line stably expressing PH-Akt-GFP, cells were transfected with the plasmid pPH-Akt-GFP using lipofectamine 2000 (Thermo Fisher). After allowing the cells to express the proteins overnight, they were trypsinized and plated sparsely in medium containing 1 mg/mL G418. After single colonies had formed, GFP-positive colonies were extracted with cloning rings. At least 6 colonies were extracted for each cell line, grown on transwell filters for 4 days, fixed, and stained against the basolateral marker protein β-catenin and the tight junction marker protein ZO-1 to assay their polarized morphology. Based on these results, we chose one colony for each cell line for further experiments.

### Caveolin-1 knockdown.

To achieve knockdown of caveolin-1 in MDCK cells, a lentivirus-based shRNA system based on the plasmids pCMV-ΔR8.91, pMD2G-VSVG, and pLVTH was used (48). The plasmid pLVTH was modified using Gibson cloning to encode the target sequence for caveolin-1 knockdown, 5′-GATGTGATTGCAGAACCAG-3′ (49). As a control, an shRNA targeted against luciferase, which is not endogenously expressed in MDCK cells, was used (target sequence, 5′-CGTACGCGGAATACTTCGA-3′). Lentivirus was produced with HEK 293 T cells, purified with sucrose cushion centrifugation (20% sucrose, 4,000 × *g*, 14 h), resuspended in MDCK medium, and applied to freshly seeded MDCK cells. To ensure a lentivirus transduction efficiency of >80%, GFP fluorescence was checked after 48 h, since pLVTH also encodes GFP. Knockdown efficiency was then verified using WB (Fig. S7).

### Immunofluorescence.

Cells were washed two times with phosphate-buffered saline without Ca^2+^ and Mg^2+^ (PBS) and then fixed with 4% (wt/vol) formaldehyde (FA) for 15 min at room temperature. Samples were treated with 50 mM ammonium chloride for 5 min to quench FA and then permeabilized with a SAPO medium (PBS supplemented with 0.2% [wt/vol] bovine serum albumin and 0.02% [wt/vol] saponin) for 30 min. Primary antibodies were diluted in SAPO medium and applied on the samples for 60 min at room temperature. After three washes with PBS, secondary dye-labeled antibodies, and, if required, DAPI (4′,6-diamidino-2-phenylindole) and dye-labeled phalloidin were diluted in SAPO medium and applied to the cells for 30 min at room temperature (details for the antibodies used are listed in Table S2). After 5 washes with PBS, cells were mounted for microscopy using glycerol-based medium supplemented with DABCO (MDCK) (50) or Mowiol-based medium (H1975) (51).

### Microscopy of fixed cells and live-cell experiments.

For imaging, an A1R confocal microscope (Nikon) equipped with a 60× oil immersion objective (numeric aperture [NA] = 1.49) and laser lines at 405 nm, 488 nm, 561 nm, and 641 nm was utilized. Image acquisition and analysis was performed with NIS-Elements 4.10.04 (Nikon).

For live-cell experiments, MDCK cells stably expressing PH-Akt-GFP (uptake of LecB-coated beads) were grown as polarized monolayers for 3 days on Lab-Tek II chambered cover glasses (8 wells, number 1.5 borosilicate glass). The medium was changed to recording medium (Hanks’ balanced salt solution [HBSS] supplemented with 1% FCS, 4.5 g/L glucose, and 20 mM HEPES).

### WB.

Before Western blotting, cells were starved in medium without FCS (16 h for polarized MDCK cells, 2 h for H1975 cells), and stimulation was also carried out in medium without FCS. After stimulation, cells were washed twice with PBS and lysed in radioimmunoprecipitation assay (RIPA) buffer (20 mM Tris [pH 8], 0.1% [wt/vol] SDS, 10% [vol/vol] glycerol, 13.7 mM NaCl, 2 mM EDTA, and 0.5% [wt/vol] sodium deoxycholate in water) supplemented with protease inhibitors (0.8 μM aprotinin, 11 μM leupeptin, 200 μM Pefabloc) and phosphatase inhibitor (1 mM sodium orthovanadate). Protein concentrations were analyzed using a bicinchoninic acid (BCA) assay kit (Pierce). Equal amounts of protein per sample were separated by SDS-PAGE and transferred to a nitrocellulose membrane. The membrane was blocked with tris-buffered saline (TBS) supplemented with 0.1% (vol/vol) Tween 20 and 3% (wt/vol) BSA for 1 h and incubated with primary and (HRP)-linked secondary antibodies diluted in the blocking solution. Detection was performed by a chemiluminescence reaction using the Fusion-FX7 Advance imaging system (Peqlab Biotechnologie GmbH). If not indicated otherwise, control samples were treated with the same volume of PBS that was used for dissolving LecB in the LecB-treated samples.

### Rac123-G-LISA.

Rac activation was measured with a Rac123-G-LISA assay (absorbance based; Cytoskeleton, Inc.) performed according to the manufacturer’s protocol. Briefly, cells were serum starved, stimulated as indicated, and then lysed. The lysates were applied to provided 96-well plates, and activated Rac was detected at 490 nm using a plate reader (Tecan Safire). If not indicated otherwise, control samples were treated with the same volume of PBS that was used for dissolving LecB in the LecB-treated samples.

### Bacterial culture and invasion assays.

For our experiments, we used GFP-tagged P. aeruginosa PAO1 wild-type (PAO1-wt) and an in-frame LecB deletion mutant (PAO1-dLecB) that were described previously (52). Bacteria were cultured overnight (approximately 16 h) in LB-Miller medium containing 60 μg/mL gentamicin in a shaker (Thriller; Peqlab) at 37°C and 650 rpm. The bacteria reached an optical density (OD) measured at 600 nm of approximately 5.

MDCK cells were allowed to polarize on transwell filters or 24-well plates as indicated. H1975 cells were cultured in 24-well plates to a confluence of 70 to 80%. Overnight cultures of PAO1-wt and PAO1-dLecB were pelleted, resuspended in DMEM (MDCK) or RPMI (H1975), and incubated for 30 min at 37°C. For inhibition with l-fucose, 100 mg/mL l-fucose was added during this incubation. The inhibitors PP2 and LY294002 were preincubated for 30 min with the cells and kept on the cells during the whole experiment. Next, the concentration of bacteria was adjusted to yield the desired multiplicity of infection (MOI) of 50. For determining the total number of bacteria, cells were incubated with bacteria for 2 h at 37°C, washed three times with PBS, and then lysed with 0.25% (vol/vol) Triton X-100. Serial dilutions of the cell extracts were made and plated on LB-Miller agar plates containing gentamicin (60 μg/mL) and incubated overnight at 37°C. The number of bacterial colonies was counted on the next day. For determining the number of invading bacteria, cells were incubated with bacteria for 2 h at 37°C and washed three times with PBS. Then, extracellular bacteria were killed by treatment with 400 μg/mL amikacin sulfate (Sigma-Aldrich) for 2 h at 37°C. After lysis with 0.25% (vol/vol) Triton X-100, bacterial numbers were counted as described before. The invasion efficiencies were calculated by dividing the number of invading bacteria by the total number of bacteria. To enable comparison between different experiments, the invasion efficiencies in a single experiment were normalized to the invasion efficiency of the untreated sample and then the mean value from repeated experiments was calculated.

### Labeling of lectins.

LecB was labeled with Cy3 monoreactive *N*-hydroxysuccinimide (NHS) ester (GE Healthcare) or with biotin using NHS-polyethylene glycol 12 (PEG12)-biotin (Thermo Fisher) according to the instructions of the manufacturers and purified using PD-10 desalting columns (GE Healthcare). StxB was labeled with NHS-ester conjugated with Alexa Fluor 488 (Thermo Fisher).

### Preparation of LecB-coated beads.

Biotinylated LecB (LecB-biotin) was incubated with a solution containing streptavidin-coated polystyrene beads containing the dye flash red with 1-μm diameter (Bangs Laboratories). To ensure homogenous coverage with LecB-biotin, a 10-fold molar excess of LecB-biotin compared to the available streptavidin binding sites on the beads was used, and then beads were washed three times with PBS. In control beads, the streptavidin binding sites were saturated with biotin.

### Mass spectrometry-based identification of LecB interaction partners.

MDCK cells were cultured in medium for stable-isotope labeling by amino acids in cell culture (SILAC medium) for 9 passages and then seeded on transwell filters and allowed to polarize for 4 days. For the first sample, biotinylated LecB was applied to the apical side of light-SILAC-labeled cells and on the basolateral side of medium-SILAC-labeled cells, whereas heavy-SILAC-labeled cells received no stimulation and served as a control. For the second sample, the treatment conditions were permuted. After lysis with immunoprecipitation (IP) lysis buffer, the different SILAC lysates were combined and LecB-biotin-receptor complexes were precipitated using streptavidin agarose beads as described before. Eluted LecB-biotin-receptor complexes were then prepared for MS analysis using SDS-PAGE gel electrophoresis. Gels were cut into pieces, the proteins therein digested with trypsin, and the resulting peptides were purified by stop-and-go-extraction (STAGE) tips. MS analysis was carried out as described previously (19) using a 1200 HPLC (Agilent Technologies, Waldbronn, Germany) connected online to a linear trap quadrupole (LTQ) Orbitrap XL mass spectrometer (Thermo Fisher Scientific, Bremen, Germany). From the list of MS-identified proteins generated, we defined those proteins as LecB interaction partners that showed more than 2-fold enrichment on a log_2_ scale over controls in both SILAC samples (Table S1).

### Statistics.

If not stated otherwise, data obtained from *n* = 3 independent experiments were used to calculate arithmetic means, and error bars represent standard errors of the means (SEM). Statistical significance analysis was carried out using GraphPad Prism 5. For determining the significance in experiments with multiple conditions, one-way analysis of variance (ANOVA) with Bonferroni’s *post hoc* testing was applied. For determining the significance in experiments in which values were measured for one condition relative to the control condition, one-sample *t* testing was applied. n.s. denotes not significant, * denotes *P* < 0.05, ** denotes *P* < 0.01, *** denotes *P* < 0.001, and **** denotes *P* < 0.0001. All primary data are available from the authors upon request.
