# Supplementary material for: The Lectin LecB Induces Patches with Basolateral Characteristics at the Apical Membrane to Promote Pseudomonas aeruginosa Host Cell Invasion
Source: mBio. 2022 May 2;13(3):e00819-22. doi: 10.1128/mbio.00819-22 (PMC9239240; doi:10.1128/mbio.00819-22)
Supplement: FIG S1 [file mbio.00819-22-s0001.docx]

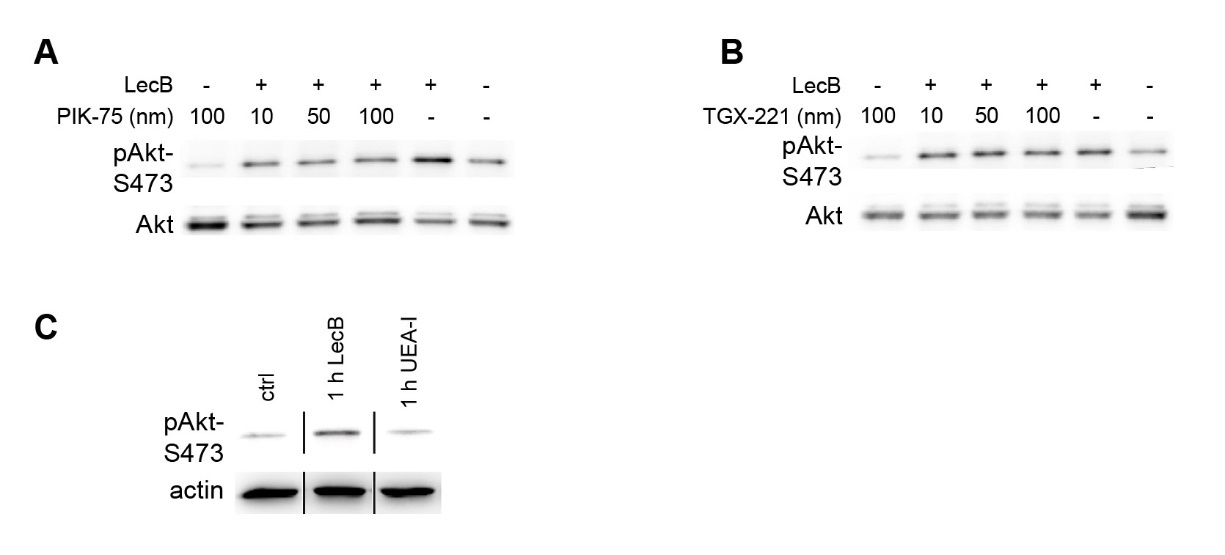


**Fig. S1: Control experiments related to Figure 1**

(A) MDCK cells were treated apically with LecB and with the indicated concentrations of the p110α-specific inhibitor PIK-75 for 1 h and Akt activation (pAkt-S473) was probed by WB analysis. (B) MDCK cells were treated apically with LecB and with the indicated concentrations of the p110β-specific inhibitor TGX-221 for 1 h and Akt activation (pAkt-S473) was probed by WB analysis. (C) MDCK cells were treated apically with 50 µg/ml UEA-I or LecB and Akt activation (pAkt-S473) was probed by WB analysis.
