# Supplementary material for: The Lectin LecB Induces Patches with Basolateral Characteristics at the Apical Membrane to Promote Pseudomonas aeruginosa Host Cell Invasion
Source: mBio. 2022 May 2;13(3):e00819-22. doi: 10.1128/mbio.00819-22 (PMC9239240; doi:10.1128/mbio.00819-22)
Supplement: FIG S2 [file mbio.00819-22-s0002.docx]

**
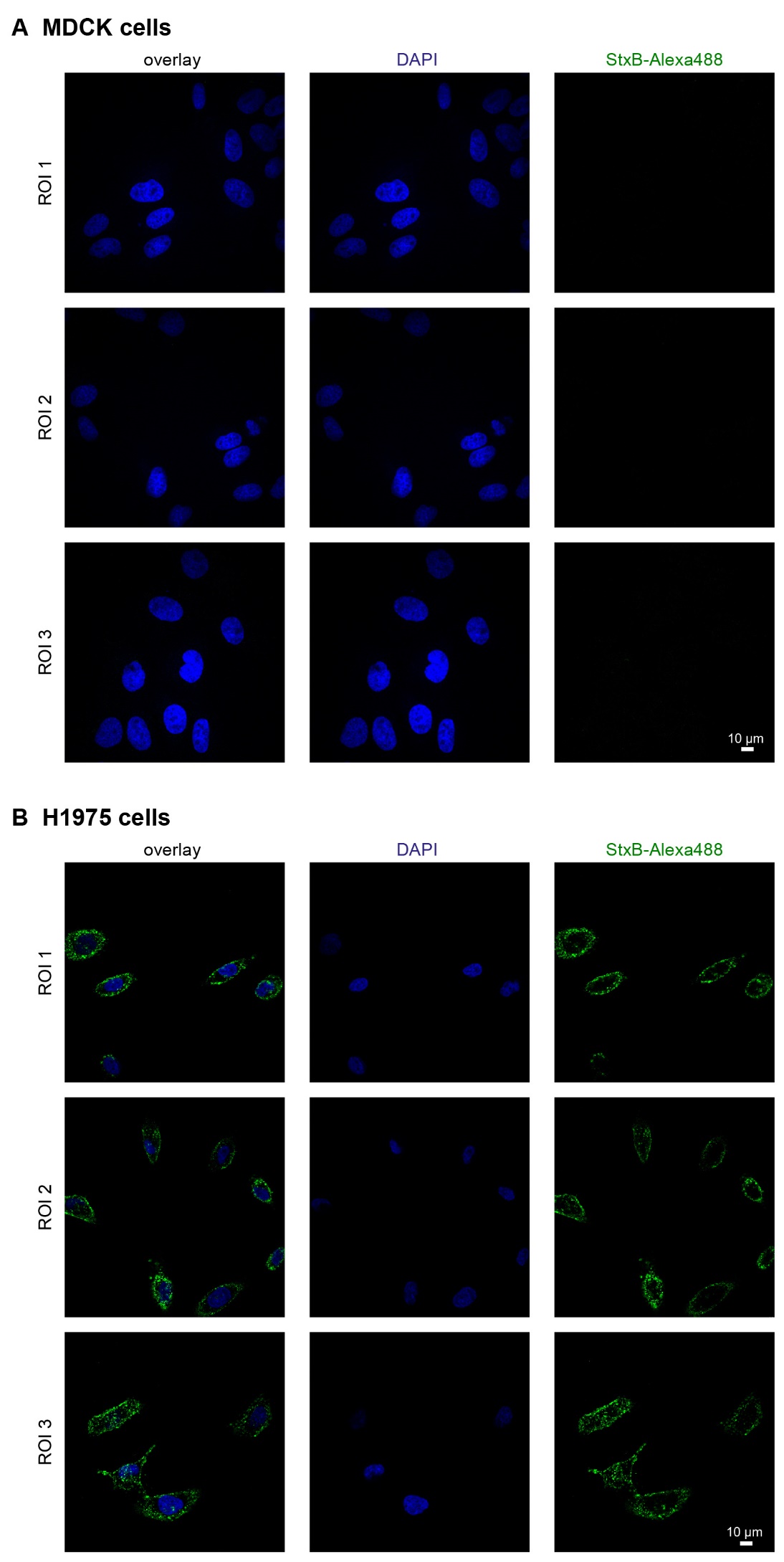
**

**Fig. S2: Evaluation of Gb3 expression in MDCK cells and H1975 cells**

MDCK cells (A) and H1975 cells (B) were seeded sparsely on glass cover slips and then incubated for 30 min at 37°C with 1 µg/ml StxB-Alexa488 (green). StxB is a lectin that specifically binds the glycosphingolipid Gb3. After fixation, cell nuclei were stained with DAPI (blue) and all samples were imaged with a confocal microscope using the same settings to ensure comparability. For each cell type, three different randomly chosen regions of interest (ROI) are displayed. Whereas MDCK cells do not bind StxB and are therefore Gb3-negative, H1975 cells show detectable binding of StxB and are hence expressing Gb3.
