# Supplementary material for: The Lectin LecB Induces Patches with Basolateral Characteristics at the Apical Membrane to Promote Pseudomonas aeruginosa Host Cell Invasion
Source: mBio. 2022 May 2;13(3):e00819-22. doi: 10.1128/mbio.00819-22 (PMC9239240; doi:10.1128/mbio.00819-22)
Supplement: FIG S3 [file mbio.00819-22-s0003.docx]

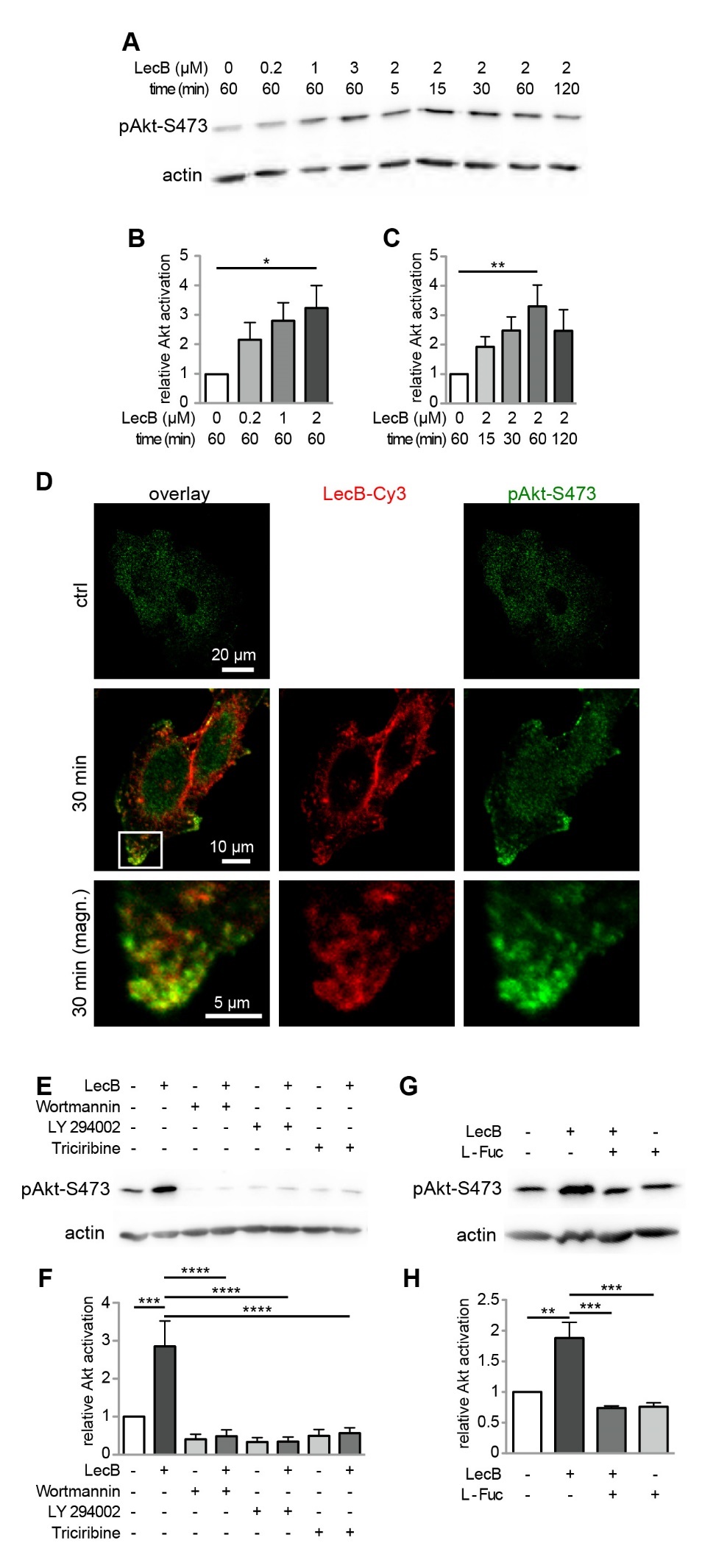


**Fig. S3: In H1975 lung epithelial cells LecB also activates PI3K/Akt signaling**

(A) – (C) H1975 cells were treated with LecB as indicated and Akt activation (pAkt-S473) was probed by WB analysis. The image depicts a representative WB; quantifications from n = 3 independent experiments for the dose-dependence and the time-dependence are depicted in (B) and (C), respectively. (D) H1975 cells were treated with LecB-Cy3 (red), fixed, and activated Akt was visualized by an antibody specific for pAkt-S473 (green). (E) – (F) H1975 cells were treated with LecB and PI3K-inhibitors Wortmannin (100 nM), LY294002 (10 µM), and the Akt inhibitor Triciribine (10 µM) for 1 h and Akt activation (pAkt-S473) was probed by WB analysis. (E) shows a representative WB, a quantification from n = 3 independent experiments is depicted in (F). (G) – (H) H1975 cells were treated with LecB and L-fucose (43 mM) for 1 h and Akt activation (pAkt-S473) was probed by WB analysis. (G) shows a representative WB, a quantification from n = 3 independent experiments is depicted in (H).
