# Supplementary material for: The Lectin LecB Induces Patches with Basolateral Characteristics at the Apical Membrane to Promote Pseudomonas aeruginosa Host Cell Invasion
Source: mBio. 2022 May 2;13(3):e00819-22. doi: 10.1128/mbio.00819-22 (PMC9239240; doi:10.1128/mbio.00819-22)
Supplement: TABLE S1 [file mbio.00819-22-s0009.docx]

**Table S1: List of apical LecB interaction partners identified by SILAC MS**

| **#** | **Protein IDs** | **Gene name** | **Number of proteins** | **Peptides** | **PEP** | **log2 (apical vs ctrl) 01** | **log2 (apical vs ctrl) 02** |
| --- | --- | --- | --- | --- | --- | --- | --- |
| 1 | J9PB47;A1YV64 | CAECAM1; CEACAM28 | 2 | 4 | 9,72E-38 | 7,24 | 6,91 |
| 2 | E2RTH3 | GGT1 | 1 | 7 | 6,34E-49 | 6,18 | 5,08 |
| 3 | Q9TSX8;F1PAX0;E2QYS4;F1PDF3 | VNN1 | 4 | 5 | 1,01E-20 | 6,11 | 3,27 |
| 4 | J9P6J1;F1Q3G6 | MUC1 | 2 | 6 | 6,91E-35 | 5,24 | 5,06 |
| 5 | F6XRM5 | ENPEP | 1 | 5 | 4,16E-36 | 5,12 | 2,88 |
| 6 | P33729;F1PB95 | ICAM1 | 2 | 7 | 6,33E-27 | 5,07 | 5,34 |
| 7 | E2QSC8 | TSPAN8 | 1 | 2 | 1,67E-29 | 4,58 | 4,27 |
| 8 | F1PU73 | NCSTN | 1 | 6 | 1,11E-23 | 4,49 | 4,81 |
| 9 | P33724;P33724-2; F1PWG1 | CAV1 | 3 | 3 | 1,89E-15 | 4,24 | 4,02 |
| 10 | F1PID4;J9P7C6;Q52S86 | PODXL | 3 | 9 | 2,71E-83 | 4,17 | 6,80 |
| 11 | F1PFS1 | STOM | 1 | 5 | 6,91E-32 | 4,03 | 3,87 |
| 12 | F1PP08;J9P8B0 | DPP4 | 2 | 15 | 1,43E-53 | 3,92 | 4,61 |
