# Supplementary material for: The Lectin LecB Induces Patches with Basolateral Characteristics at the Apical Membrane to Promote Pseudomonas aeruginosa Host Cell Invasion
Source: mBio. 2022 May 2;13(3):e00819-22. doi: 10.1128/mbio.00819-22 (PMC9239240; doi:10.1128/mbio.00819-22)
Supplement: FIG S4 [file mbio.00819-22-s0004.docx]

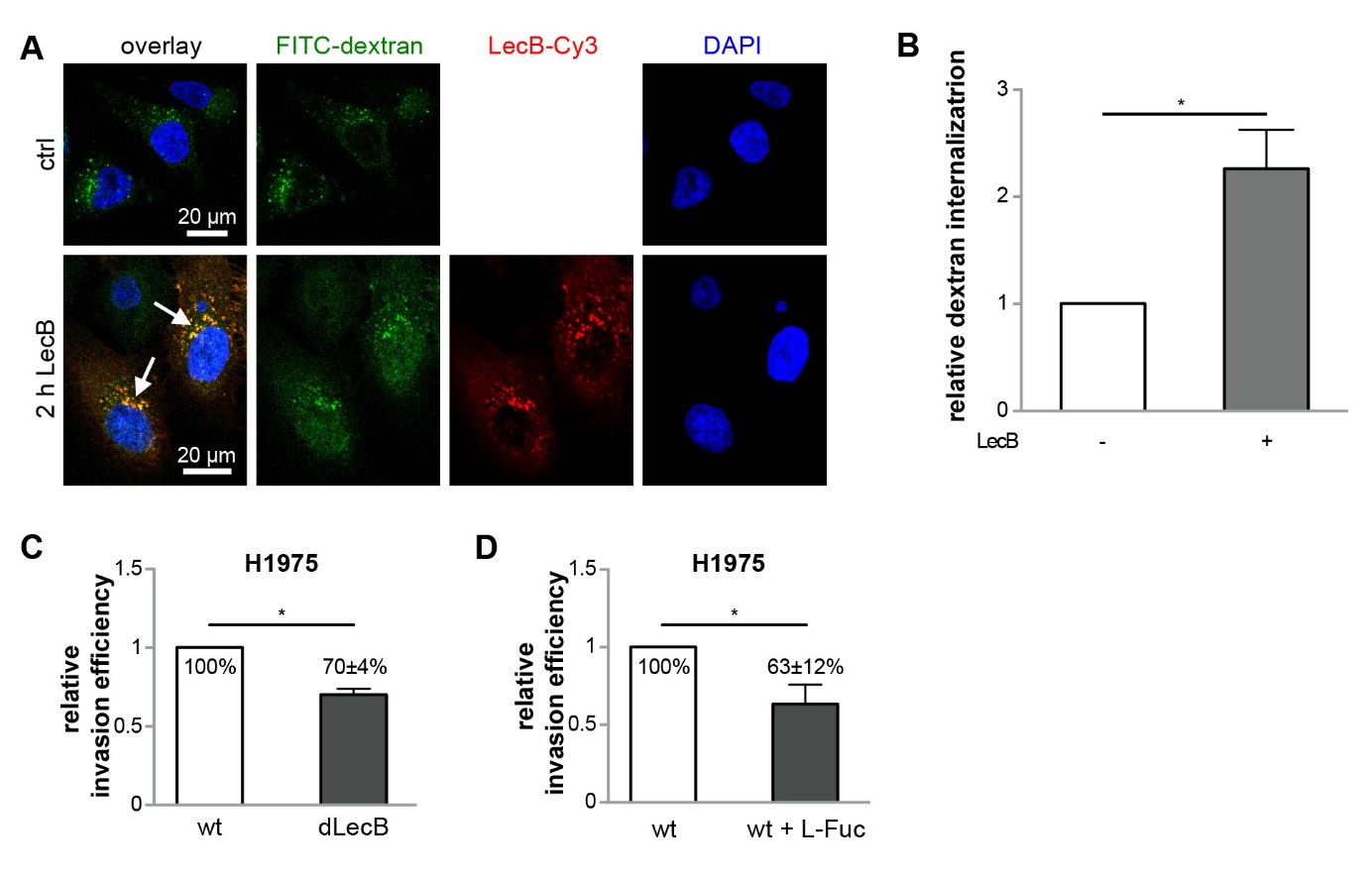


**Fig. S4: LecB stimulates macropinocytosis and facilitates *P. aeruginosa* invasion in H1975 cells**

(A) – (B) Representative images of untreated H1975 cells and cells treated with LecB-Cy3 for 2 h (red). To probe macropinocytosis cells were also incubated with 0.2 µM FITC-dextran (green); nuclei were stained with DAPI (blue). White arrows point to internalized FITC-dextran co-localizing with LecB-Cy3. (D) Quantification of FITC-dextran uptake in untreated and LecB-treated cells; n = 3. (C) – (D) Amikacin protection assays measuring the invasion of PAO1-wt and PAO1-dLecB (C) and PAO1-wt pre-incubated with 100 mg/ml L-fucose (D) in H1975 cells. Invasion for 2 h, MOI 50, n = 3.
