# Supplementary material for: The Lectin LecB Induces Patches with Basolateral Characteristics at the Apical Membrane to Promote Pseudomonas aeruginosa Host Cell Invasion
Source: mBio. 2022 May 2;13(3):e00819-22. doi: 10.1128/mbio.00819-22 (PMC9239240; doi:10.1128/mbio.00819-22)
Supplement: FIG S5 [file mbio.00819-22-s0005.docx]

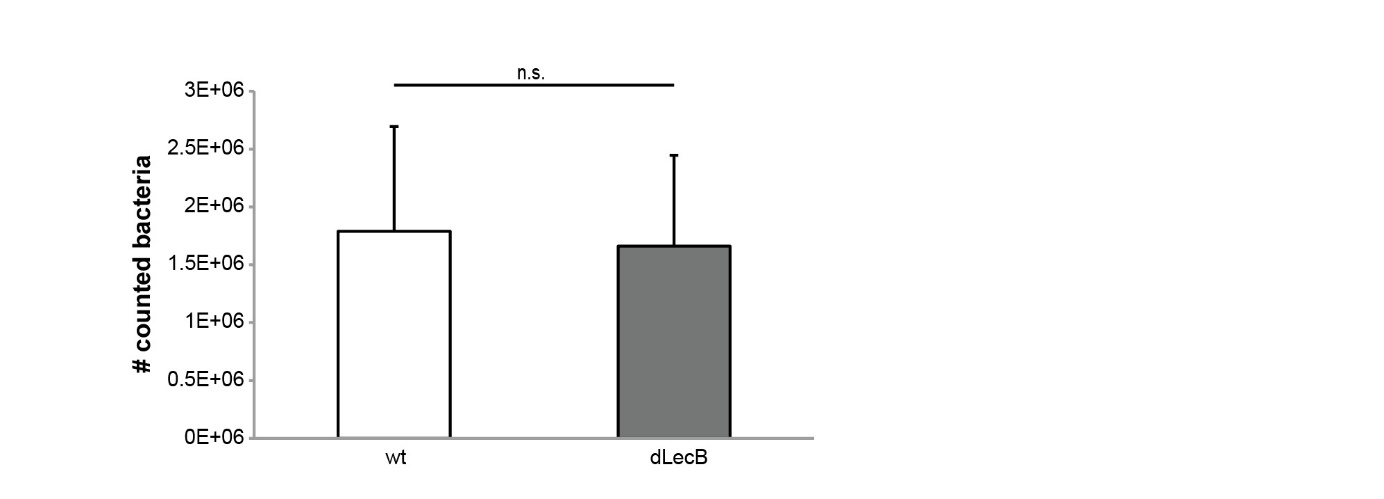


**Fig. S5: Comparison of cell association of wt and dLecB *P.aeruginosa***

Wt and dLecB *P. aeruginosa* were applied to polarized MDCK cells at MOI 50 for 2 h. Afterwards, cells were lysed with 0.25% (v/v) Triton X-100. Serial dilutions of the cell extracts were made and plated on LB–Miller agar plates containing gentamicin (60 μg/ml) for counting. This corresponds to the procedure for determining the total number of bacteria in the amikacin protection assays. The graph shows the mean values of counted bacteria from n = 8 experiments.
