# Supplementary material for: The Lectin LecB Induces Patches with Basolateral Characteristics at the Apical Membrane to Promote Pseudomonas aeruginosa Host Cell Invasion
Source: mBio. 2022 May 2;13(3):e00819-22. doi: 10.1128/mbio.00819-22 (PMC9239240; doi:10.1128/mbio.00819-22)
Supplement: FIG S6 [file mbio.00819-22-s0006.docx]

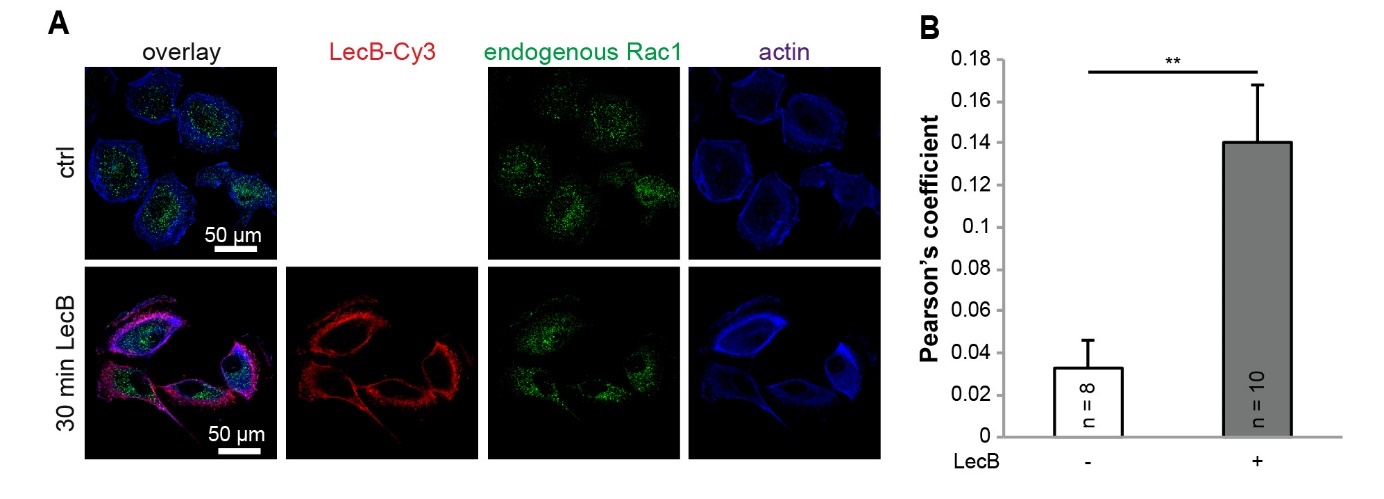


**Fig. S6: Control experiment related to Figure 3**

(A) – (B) H1975 cells were treated with LecB-Cy3 (red) as indicated, fixed, and stained for actin with phalloidin-Atto647 (blue) and endogenous Rac1 (green) and then imaged with a confocal microscope. (A) Representative images. (B) The Pearson’s co-localization coefficient between endogenous Rac1 and actin was determined in individual cells and the average was calculated.
