# Supplementary material for: The Lectin LecB Induces Patches with Basolateral Characteristics at the Apical Membrane to Promote Pseudomonas aeruginosa Host Cell Invasion
Source: mBio. 2022 May 2;13(3):e00819-22. doi: 10.1128/mbio.00819-22 (PMC9239240; doi:10.1128/mbio.00819-22)
Supplement: FIG S7 [file mbio.00819-22-s0007.docx]

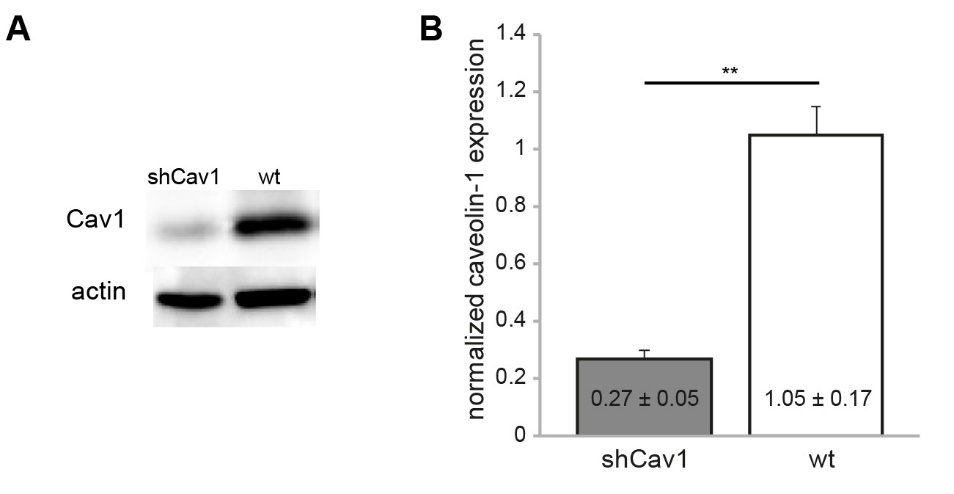


**Fig. S7: Verification of caveolin-1 knockdown in shCav1 cells**

(A) – (B) The expression of caveolin-1 in caveolin-1 knockdown MDCK cells (shCav1) and wild type MDCK cells (wt) were assessed by WB analysis. (A) Representative WB. (B) Quantification of caveolin-1 knockdown from n = 3 independent experiments. To calculate the normalized caveolin-1 expression, the caveolin-1 band intensities were divided by actin band intensities for each individual sample and averaged. Error bars represent SEM, statistical significance was evaluated by a paired two-sided t-test, ** denotes p <0.01.
