# Supplementary material for: The Lectin LecB Induces Patches with Basolateral Characteristics at the Apical Membrane to Promote Pseudomonas aeruginosa Host Cell Invasion
Source: mBio. 2022 May 2;13(3):e00819-22. doi: 10.1128/mbio.00819-22 (PMC9239240; doi:10.1128/mbio.00819-22)
Supplement: TABLE S2 [file mbio.00819-22-s0010.docx]

**Table S2: Lists of used primary and secondary antibodies** (WB … Western Blot, IF … immunofluorescence)

| *Primary antibodies:* |  |  |  |  |
| --- | --- | --- | --- | --- |
| **antibody target** | **generated in species** | **supplier** | **product number** | **dilution (application)** |
| acetylated tubulin | mouse | Sigma Aldrich | T7451 | 1:3000 (IF) |
| Akt (pan) | mouse | Cell Signaling | 2920 | 1:1000 (WB) |
| p-PI3K p85-Y458 /  p55-Y199 | rabbit | Cell Signaling | 4228 | 1:100 (IF);  1:1000 (WB) |
| pAkt-S473 | rabbit | Cell Signaling | 4060 | 1:100 (IF);  1:1000 (WB) |
| caveolin-1 | rabbit | Cell Signaling | 3267 | 1:250 (IF);  1:1000 (WB) |
| Rac1 | mouse | Cytoskeleton | ARC03 | 1:100 (IF) |
| β-actin | mouse | Sigma Aldrich | A5316 | 1:1000 (WB) |
| β-catenin | rabbit | Abcam | ab32572 | 1:250 (IF) |
| β1-integrin | mouse | Millipore | MAB2000 | 1:200 (IF) |
| ZO-1 | rat | Millipore | MABT11 | 1:50 (IF) |
| *Secondary antibodies:* |  |  |  |  |
| **antibody target** | **conjugation** | **supplier** | **product number** | **dilution (application)** |
| anti-mouse | HRP | Cell Signaling | 7076 | 1:2000 (WB) |
| anti-mouse | Alexa488 | Thermo Fisher | A21202 | 1:200 (IF) |
| anti-mouse | Cy3 | Jackson Immuno-research | 715-166-1500 | 1:200 (IF) |
| anti-mouse | Alexa647 | Thermo Fisher | A21236 | 1:200 (IF) |
| anti-rabbit | HRP | Cell Signaling | 7074 | 1:2000 (WB) |
| anti-rabbit | Alexa488 | Thermo Fisher | A21206 | 1:200 (IF) |
| anti-rabbit | Cy3 | Jackson Immuno-research | 711-166-152 | 1:200 (IF) |
| anti-rabbit | Alexa647 | Thermo Fisher | A21245 | 1:200 (IF) |
| anti-rat | Alexa488 | Thermo Fisher | A21208 | 1:200 (IF) |
